# Shifts in Climate Foster Exceptional Opportunities for Species Radiation: The Case of South African Geraniums

**DOI:** 10.1371/journal.pone.0083087

**Published:** 2013-12-17

**Authors:** Hugo I. Martínez-Cabrera, Pedro R. Peres-Neto

**Affiliations:** 1 Département des sciences biologiques, Université du Québec à Montréal, Montreal, Quebec, Canada; 2 Canada Research Chair in Spatial Modelling and Biodiversity, Université du Québec à Montréal Montreal, Quebec, Canada; University of Kent, United Kingdom

## Abstract

Climate change is often assumed to be a major driver of biodiversity loss. However, it can also set the stage for novel diversification in lineages with the evolutionary ability to colonize new environments. Here we tested if the extraordinary evolutionary success of the genus *Pelargonium* was related to the ability of its species to capitalize on the climate niche variation produced by the historical changes in southern Africa. We evaluated the relationship between rates of climate niche evolution and diversification rates in the main *Pelargonium* lineages and disentangled the roles of deep and recent historical events in the modification of species niches. *Pelargonium* clades exhibiting higher ecological differentiation along summer precipitation (SPP) gradients also experienced higher diversification rates. Faster rates of niche differentiation in spatially structured variables, along with lower levels of niche overlap among closely related species, suggest recent modification in species niches (e.g. dispersal or range shift) and niche lability. We suggest that highly structured SPP gradients established during the aridification process within southern Africa, in concert with niche lability and low niche overlap, contributed to species divergence. These factors are likely to be responsible for the extensive diversification of other lineages in this diversity hot spot.

## Introduction

Patterns of species diversification associated with past climate shifts can offer insights into the processes generating biological diversity and may provide information on how lineages are likely to respond to future climate change. Under changing climate, lineages with rapidly evolving climatic preferences may have higher diversification success [1] because they are more likely to colonize and spread into new climate zones [2]. We now know that this capacity is mainly determined by the extent to which their climate niche is evolutionarily conserved [3] (but see [4], [5] for exceptions), so that if phylogenetic niche conservatism is strong, species will probably fail to adapt and thus experience greater risks of extinction if the new environments are different from their ancestral state. When a new selective regime is established (e.g. new climate regime), niche lability may fuel species diversification [6] (but see [7]) by increasing the rate at which niche dimensions associated with the new regime evolve [3], [6]. Niche conservatism, on the other hand, might hinder evolution of climate niche preferences even if change in climate has increased ecological opportunities for diversification [3].

Phylogenetic niche conservatism among closely related species originates due to phylogenetic [5], [8] or spatial constraints [8], [9] or a combination of both. This is because both, inherited environmental preferences due to common ancestry or dispersal constraints in relation to the ancestral range, can shape the opportunities for adaptation to new environments [8]. Spatial and phylogenetic components of niche variation may be equally important if they are intrinsically related (i.e. spatial distances among species have a strong relationship with their phylogenetic history). When the spatial component of niche variation is greater than the effect of phylogeny, it is suggested that species responses to recent evolutionary events were more important than deeper historical events for determining species niches [8]. For instance, Cooper et al. [10] found that the large spatial component of mammal niche variation could be mostly explained by a modification in species niches that evolved in response to recent environmental changes - possibly related to dispersal or shifts in geographical ranges.

Under rapid climate change, and particularly during the rapid establishment of seasonal gradients, ancestral ranges likely undergo climatic fragmentation (i.e. different populations occupy different climate zones in the new regime). This would cause reproductive isolation and species divergence and result in closely related species occupying distinct portions of the climate niche space. If true, then climate niche axes that have strong spatial signatures would also show higher rates of evolution because closely related species would show divergence in climate preferences. It follows that lineages exhibiting high niche lability would be expected to diversify more rapidly than lineages with highly conserved climate preferences, which are also more likely to go extinct. Due to such niche segmentation of the ancestral ranges, closely related species within lineages diversifying during the establishment of climate gradients should then also exhibit low degrees of niche overlap.

In the Cape Floristic Region (CFR) of South Africa, the rapid climate change which lead to the aridification and establishment of the Mediterranean type climate started during the Miocene and continued until the Pleistocene, and was driven by both changes in the position of high-pressure cells and the extent of the Antarctic ice sheet [11], [12]. The subsequent widespread extinction of the western South Africa flora [13], [14] is thought to have created a significant amount of vacant niches space and additional opportunities for diversification [15], [16] among lineages adapted to summer aridity [14]. This climate change process increased niche availability in this system in two ways: 1) the establishment of a seasonal precipitation gradient with winter rainfall in the west *versus* summer rainfall in the east; and 2) within the winter rainfall regime, a sharp gradient in winter rainfall amounts [17], [18]. The rapid establishment of climate gradients in highly segmented landscapes [19] would likely accelerate ecological differentiation among species within lineages showing climate niche lability. The wide range of habitats occupied by *Pelargonium*'s closely related species [20], [21], as well as their diverse morphological adaptations [22], suggest that indeed this group has adaptively radiated [23]. This niche filling process in an adaptive radiation may lead to an acceleration in the accumulation of lineages, though as niche space becomes filled diversification rates may actually decline [6], [24], [25].

In this study we set out to determine the extent to which ecological differentiation has occurred in the genus and to know if this was associated with historical shifts in climate regimes. We also analyzed whether *Perlargonium* clades experiencing accelerated evolution of climate preferences and high species diversity underwent declines in diversification rates associated with the saturation of the climate niche space. Lastly, we explored the relationship between niche diversification rates, niche overlap and the relative importance of space *versus* phylogeny in determining climate niche structure. In particular, we tested whether those niche dimensions over which the genus had experienced greater ecological differentiation also exhibited greater spatial structure and low niche overlap. We expect that clades experiencing successful radiations would be associated with a greater ability to explore the newly established climatic regimes (niche lability). As a consequence, the rates of evolution of climate niche preferences should be positively related with clade diversification rates and richness.

## Materials and Methods

### Phylogeny, species distributions and environmental coverage

Rate diversification and comparative analyses were based on Bakker et al.'s 50% majority rule Bayesian consensus tree [26]. This phylogenetic hypothesis is based on four data partitions: cpDNA *trn*L-F, nrDNA ITS, mtDNA *nad*1b/c, and 30 recoded indels inferred from the plastid partition [22], [26]. For the analysis of niche evolution, we pruned this phylogenetic tree (originally containing 150 species) to include only the 112 species with distributional information; distribution was obtained from digital databases [27], [28], taxonomic monographs and our collection records. In total, we analyzed over 6500 occurrence points for 112 species (Table S1), representing roughly 40% of the species in the genus. Outlier occurrences for each species (i.e. those occurrences clearly beyond the distribution of the rest of the species occurrence data) were excluded from our analyses. The resultant pruned phylogeny was used to estimate a chronogram using penalized likelihood [29] in APE [30] assuming a root age of 30 MYR corresponding to the divergence time calculated based on the ancestral divergence of Geraniaceae [13].

Climate information at 1 arc minute resolution (1.55×1.85 km at South Africa latitude) was acquired from the South African Atlas of Climatology and Agrohydrology [31]. From an initial set of 33 climate variables, 10 were selected for use based on their loadings on the first three principal components and were relevant to the niche evolution and distribution of *Pelargonium* [21]: mean annual precipitation (mm), winter and summer precipitation (mm), annual heat units (°d), accumulated positive chill units (CU), winter and summer solar radiation (MJ · m^−2^ · d^−1^), and winter and summer vapor pressure deficit (kPa).

### Diversification analyses and rates of climate preference evolution

**Figure 1:**
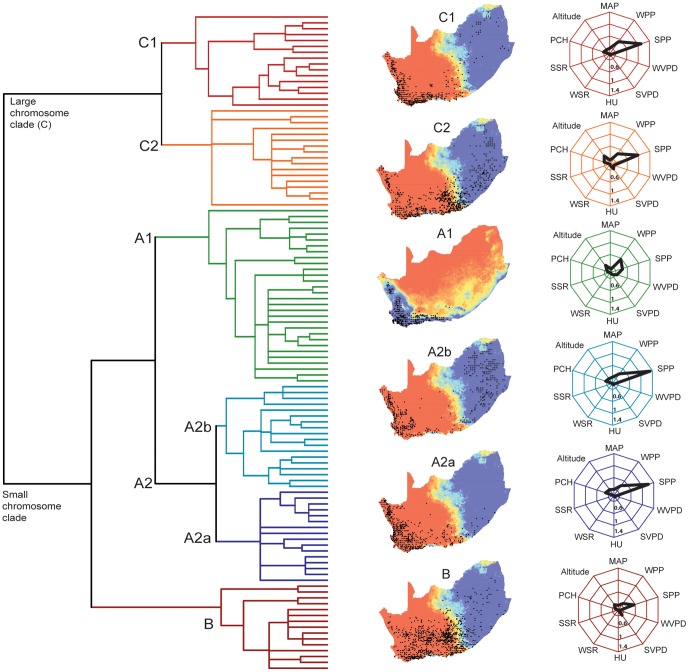
Chronogram, geographic distribution and climate rates of evolution for *Pelargonium* clades. Background colors of the maps represent summer precipitation, except for clade A1, which portrays winter precipitation. Blue and red regions represent high and low precipitation respectively. Radar plots show the rate of evolution in felsens for each climate variable in each clade; values closer to the center of the web indicate slower rates of evolution while larger β values indicate higher rates. For the actual values see Table S2. Major clades are colored in the phylogeny and radar plots. MAP =  mean annual precipitation; WPP =  mean winter precipitation; SPP =  mean summer precipitation; WVPD =  mean winter pressure deficit; SVPD =  mean summer pressure deficit; HU =  annual heat units; WSR =  mean winter solar radiation; SSR =  mean summer solar radiation; PCH =  accumulated positive chill units.

We estimated net diversification rates (speciation minus extinction) for each major clade (Fig. 1; A1, A2a, A2b, B, C1 and C2) using Magallón and Sanderson's method accounting for incomplete sampling [32] as implemented in the Geiger package for R [33]. We calculated diversification rates using three levels (*e* = 0, 0.5 and 0.99) of extinction fractions (e =  speciation/extinction), which dictate the probability of survival to the present. As clades in the large (A1, A2a, A2b and B) and small (C1, C2) chromosome clades differ in diversity and climate preferences, we tested for differences in diversification rates using the Mann-Whitney U-test. One of the consequences of rapid initial diversification is the potential for decreasing diversification rates due to species accumulation in clades [24], [34]. Since *Pelargonium* and other cape lineages (e.g. *Muraltia*, some *Restionaceae* and *Satyrium* [14]) experienced explosive *in situ* radiation, we tested whether there was a decrease in diversification rates due to species accumulation or whether, despite rapid occupation of niches, *Pelargonium* experienced constant diversification rates. To test whether diversification rates decreased or increased over time the γ statistic [35] was used. A negative γ indicates a decrease in diversification rate relative to a constant diversification model through time. Given that incomplete phylogenetic sampling is known to bias γ [35], we conducted a Monte Carlo Constant Rate Test (MCMCR test) to account for incomplete sampling. We simulated 10 000 complete phylogenies (for each clade) under the null hypothesis of constant pure birth diversification; tips were randomly pruned to conform to actual clade sizes. The critical γ value (the 0.05 percentile of the null distribution) of the simulated replicates was contrasted with the observed γ value [35], with greater observed γ values indicating a rate decrease greater than expected by chance. This analysis was run using the R package LASER [36].

We also assessed which rate diversification model best fit the data for each clade using likelihood methods [36]. We explored two rate constant (RC) models, namely pure birth and birth-death models, for inferring diversification rates that do not vary over time [36], [37], and four rate variable (RV) models: exponential (DX), linear (DL) density dependent models of diversification and two multi-rate models (Yule-2 rate and Yule-3) based on a variant of the Yule process [36]. To fit the diversification parameters, we used the ages estimated by Bakker et al. [13] for the genus (30 MY) and its main clades. To test whether the null hypothesis of the RC model could be rejected in support of an RV model, we estimated differences in fit using the Akaike Information Criterion as ΔAIC_RC_ =  AIC_RC_−AIC_Rv_ [36].

The rates of climate preference evolution for each clade were calculated using the species mean value for each climate variable. Climate evolution rates for the clades considered here were calculated using felsens [38], which is a contrast-based metric that uses the time-calibrated phylogeny and ln-tranformed variables (here climatic variables). A felsen (β, the Brownian motion rate parameter) is an increase of one unit per million years in the trait (or niche in this case) variance among sister taxa [38]. Felsens represent the PIC variance describing the rate of divergence in the climate niche variables among species.

We used regression to test the hypothesis that highly diverse clades have higher rates of diversification and to determine if highly diverse, rapidly evolving clades experience declining diversification rates, possibly as a product of niche filling. Finally, using least-squares and polynomial regression, we explored whether mean clade evolution of individual climate niche preferences is related to clade diversification rates.

### Space vs. phylogenetic signal in climate niche variables

For each clade, we evaluated the relative contribution of phylogenetic relationships versus spatial variation on evolution climate preferences across species using the Freckleton and Jetz's framework [8]. Their method modifies the expected variance of the phylogenetically independent contrast to simultaneously include a spatial and a phylogenetic signal. The relative contributions of the phylogenetic and spatial effects are estimated by *Ø*, which ranges in value between 0 (only phylogenetic structure is important) and 1 (only spatial structure is important). Based on Pagel's λ [39] and *Ø*, we calculated the spatially corrected version of Pagel's λ (λ′) and the proportion of climate variation that is not explained by space or phylogeny (Γ). Pagel's λ [39] is a branch length transformation as it is simply a multiplier of the off-diagonal elements of the variance-covariance matrix that best fits the data [8], [10]. λ measures phylogenetic signal where a value of 0 indicates no phylogenetic signal, 1 trait variation according to the Brownian motion model and a value of >1 indicates very strong phylogenetic conservatism. We can estimate the spatially corrected version of Pagel's λ as λ′ = (1−*Ø*) λ), which measures the relative contribution of phylogeny independent of space, while Γ = (1−*Ø*)(1−λ) measures the proportion of trait variation that is neither explained by space nor phylogeny [8]. We estimated *Ø* and λ for each climate variable separately.

### Niche overlap

Niche overlap among closely related species serves as a proxy for niche conservatism [40] and may explain constraints in species distributions. For each clade, we quantified species overlap for each climate variable using the metric D [41]. D varies between 0 and 1 indicating no overlap and identical niches, respectively. As there may be differences in sampling effort or design among regions, sampled occurrences may not characterize the entire range of climate suitable for species, which in turn may bias measures of niche overlap [42]. To avoid these biases, here we used the framework proposed by Broennimann et al. [42] which uses kernels to smooth density probability distributions of species occurrences in the environmental space. Since this method also uses smoothed densities of available environments, the niche overlap test within this framework compares the occurrence densities between species occurring in ranges where environments are not equally available [42]. In addition, the use of a kernel density estimator provides an unbiased estimate of D because it ensures independence between the environmental overlap and grid resolution [42]. The equivalency test [40] was used to test for significance in overlap for each environmental variable. The test consists of pooling the occurrence of any given species pair (pseudoreplicate datasets) and randomly partitioning it into two data sets (keeping the original occurrence size of the two species). This procedure was repeated for 1000 pseudoreplicate datasets and for each one, the niche overlap metric D was calculated to obtain the null distribution of similarity values. The observed value of D was then compared with its null distribution using a one-tailed test and the null hypothesis of niche equivalency was rejected if the observed D fell outside of the 95% of the simulated values, thus indicating niche differentiation.

## Results

### Diversification rates

Lineages within the small chromosome clade (A1, A2a, A2b and B) experienced higher net diversification rates (Table 1) compared to the lineages in the large chromosome clade (C1 and C2). These trends were robust across the three different extinction fractions tested (*e* = 0, 0.5 and 0.99). Net diversification rates were highest in clade A2b (*r_0_* = 0.248, *r_0.5_* = 0.229, *r_0.99_* = 0.039 speciation events per MY) and A2a (*r_0_* = 0.186, *r_0.5_* = 0.169, *r_0.99_* = 0.018), both in the xerophytic clade A2. In clades C1 (*r_0_* = 0.093, *r_0.5_* = 0.084, *r_0.99_* = 0.007) and C2 (*r_0_* = 0.099, *r_0.5_* = 0.089, *r_0.99_* = 0.007) diversification rates were the lowest. There was a marginally significant difference in diversification rates between small and large chromosome clades (*P* = 0.06, Mann-Whitney U-test). Declining diversification rates in all clades was suggested by negative values for the γ-statistic (except clade A1; Table 1). The steepest decline was detected in clade A2b (γ = −2.99, P = 0.006). Despite the negative values of γ for most clades, the null hypothesis of rate constancy, when accounting for an incomplete taxonomic sample, was only rejected for clade A2b (Table 1). In the rate variation analysis (Table S2), we found that RC models were rejected in favor of an RV model (ΔAIC_RC_ = 7.23, DL) for clade A2b only. Positive ΔAIC_RC_ in clades A1 (ΔAIC_RC_ = 0.73, Yule-2 rate), B (ΔAIC_RC_ = 0.27, DL) and C2 (ΔAIC_RC_  = 1.51, Yule-3 rate) indicate rate variation. However, for small phylogenies, the RC models can only be rejected with confidence when ΔAIC_RC_ is close to 4 [36] and as such, decreases in these last three clades cannot be asserted. In clades A2a (ΔAIC_RC_ = −0.52) and C1 (ΔAIC_RC_ = −1.75), RV models were rejected in favor of RC models (pure birth in both cases, Table S2). For the entire genus, however, there is clear evidence of rate variation (ΔAIC_RC_ = 5.83, Yule-3 rate).

**Table 1:** Results of the diversification rate analyses for *Pelargonium* clades.

	Net diversification rate			Gamma					Clade diversity	Clade age (MY)
Clade	*r_0_*	*r_0.5_*	*r_0.99_*	Observed γ	P-value	Simulated critical value γ	Observed- simulated γ	MCMCR corrected P-value		
A1	0.155	0.142	0.017	0.81	0.79	−2.06	2.88	0.91	34	20
A2a	0.186	0.169	0.018	−0.47	0.31	−1.32	0.85	0.23	20	15
A2b	0.248	0.229	0.039	−2.99	0.001	−2.96	−0.03	0.047	83	10
C1	0.093	0.084	0.007	−0.01	0.49	−1.76	1.74	0.57	23	26
C2	0.099	0.089	0.007	−0.44	0.32	−1.73	1.28	0.38	23	24
B	0.109	0.097	0.007	−0.8	0.2	−2.05	1.25	0.37	27	20

*r_0_*, *r_0.5_*, *r_0.99_*. =  net diversification rates with extinction fraction of 0, 0.5 and 0.99 respectively. MCMCR =  Monte Carlo Constant Rate Test.

### Rates of climate evolution

Mean rates of climate preference evolution varied from β = 0.25 felsens in clades A2a and A2b to β = 0.15 felsens in clade B based on all climate variables considered here (Table S3). As predicted, in most cases, rates related to precipitation variables were one order of magnitude greater than for the other climatic variables. Strikingly, even in clades mainly distributed in the winter rainfall region (A2a, A2b and C1, except for the small clade *Magnistipulacea* in clade A2a, that has radiated from the winter rainfall to the summer rainfall region, see Fig 1), the rate of evolution of summer precipitation preferences was faster than that for winter precipitation. Only in the evergreen shrubs of clade A1 (Fig. 1) winter precipitation preferences evolved faster than summer precipitation preferences. Rates for the evolutionary preference for summer precipitation observed the highest values in the winter rainfall clades A2a and A2b with β = 1.21 and 1.31 felsens, respectively. Preferences along seasonal solar radiation and vapor pressure deficit gradients evolved consistently slower across clades (Table S3, Fig. 1).

### Spatial vs. phylogenetic effects

Across all clades and for all climate variables, the importance of the spatial component *Ø* was greater than the phylogenetic component λ′, indicating that spatially-related processes and not shared phylogenetic histories were most important in driving climate niche variation. Mean clade independent variation, Γ, was generally greater than λ′ and lower than *Ø*. In most clades, variation in summer precipitation observed Ø values closest to 1, followed by summer solar radiation and mean annual precipitation (Table S4). The spatial component of summer precipitation was consistently high throughout all clades (λ′<*Ø*). Phylogenetic effects were only greater than spatial effects (λ′>*Ø*) in niche variables related to environmental energy (heat units and seasonal solar radiation) in clades A1, A2a and A2b.

### Niche Overlap

**Figure 2:**
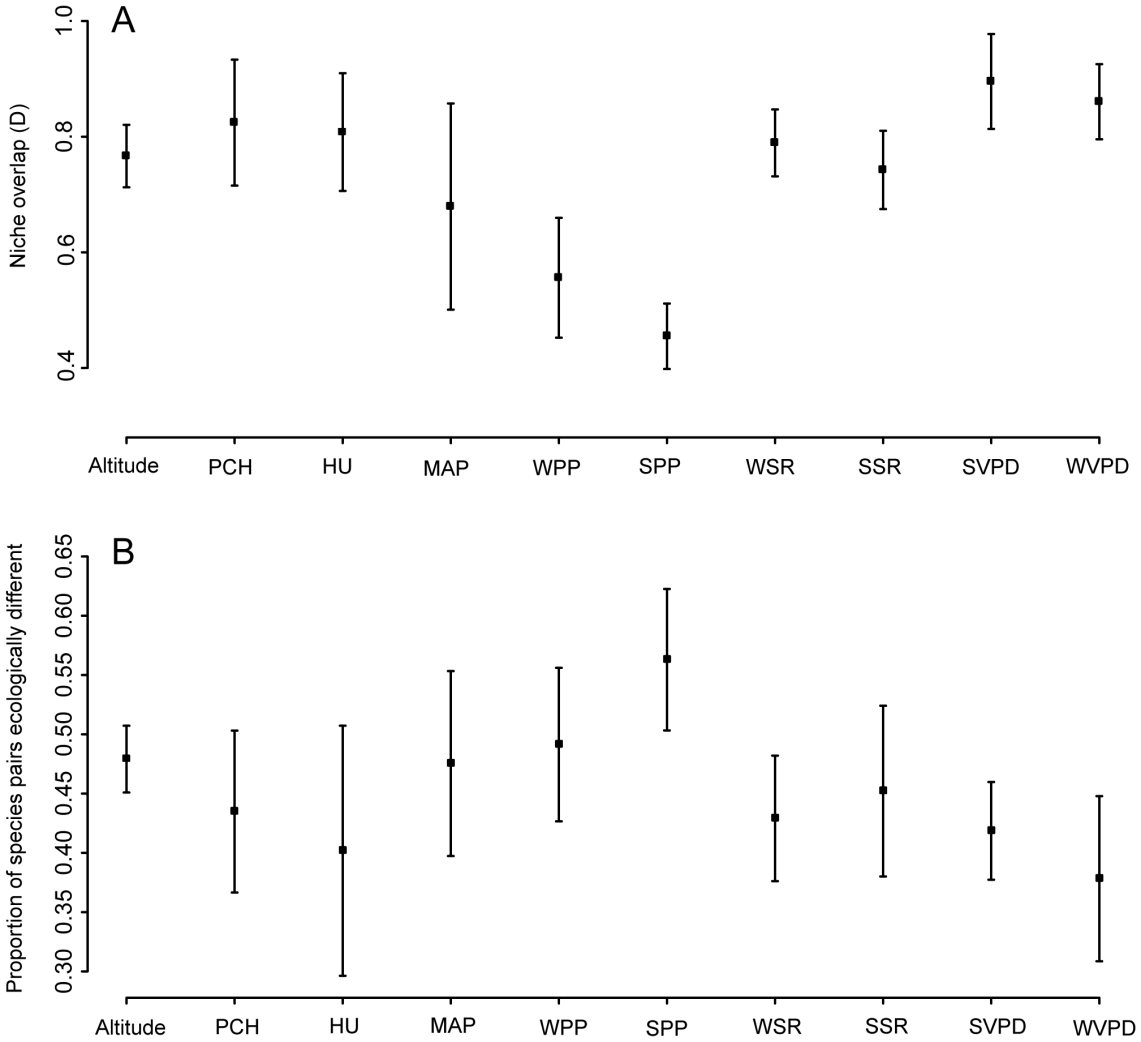
Results of the climate niche overlap analysis. The graphs show the mean and 95% confidence intervals across clades of the 2A) mean within clade pairwise species niche overlap and its 2B) proportion of rejected niche equivalency tests (proportion of species pairs ecologically distinct). D =  Schoener niche overlap metric; PCH =  accumulated positive chill units; HU =  annual heat units; MAP =  mean annual precipitation; WPP =  mean winter precipitation; SPP =  mean summer precipitation; WSR =  mean winter solar radiation; SSR =  mean summer solar radiation; WVPD =  mean winter pressure deficit; SVPD =  mean summer pressure deficit.

We used climate niche overlap among closely related species as a proxy for climate conservatism. Smaller overlaps were observed regarding summer and winter precipitation (Fig. 2A) than compared to any other climate variable. Interestingly, even for clades primarily distributed in the winter rainfall region (A1, A2a, A2b), closely related species overlapped least in their preferences for summer precipitation levels. This result strongly supports our finding that closely related species experienced large ecological differentiation (i.e. higher β) along precipitation gradients. Conversely, variables related to environmental energy such as solar radiation and vapor pressure deficit (Fig. 2A; Figure A in Figure S1) observed much greater levels of overlap and hence varied much less among closely related taxa. In all clades except A1, a larger number of pair-wise equivalence tests were rejected for precipitation variables (summer, winter or total precipitation) indicating that most of the ecological differentiation among closely related species has occurred along these niche axes (Fig. 2B; Figure B in Figure S1).

### The drivers of the rate of climate preference evolution

**Figure 3:**
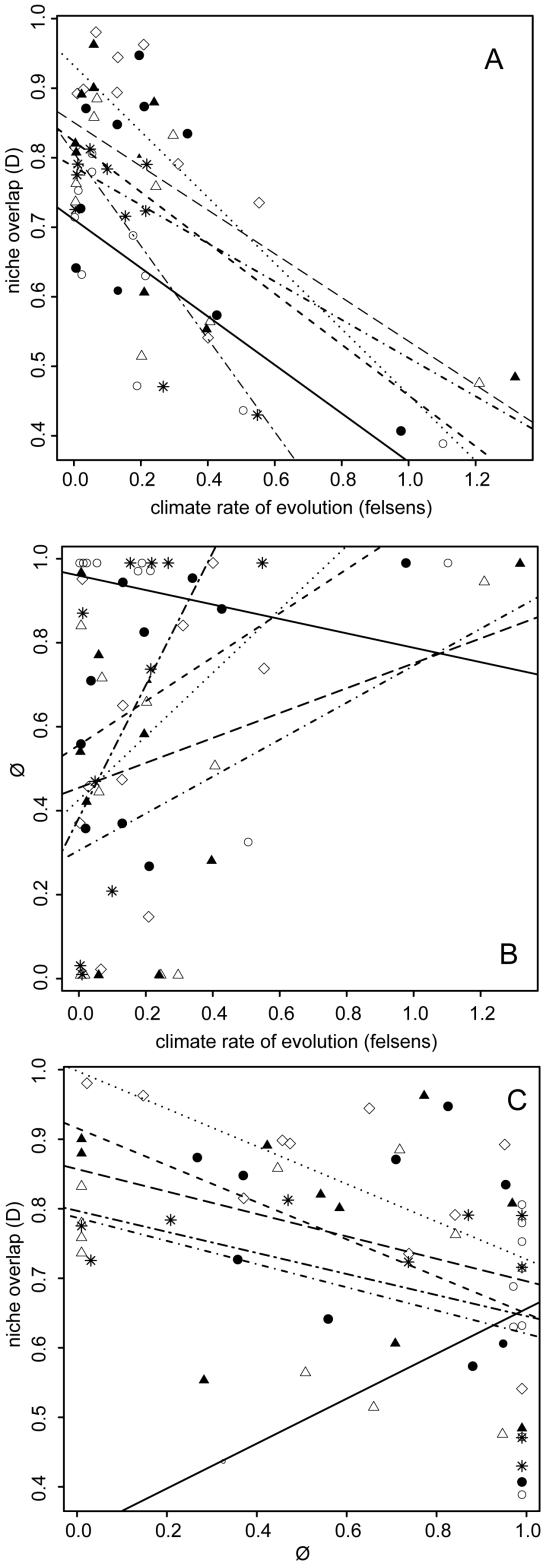
Plots showing a) niche overlap and b) *Ø* as a function of climate rate of evolution and c) between niche overlap and *Ø* for each of the *Pelargonium* clades. Each regression line represents a clade; data points are climate variables for each clade. Open circles and solid line  =  clade C1; filled circle and dashed line  = C2; open triangles and dot-dashed line  = A2a; closed triangles and long-dashed line  = A2b; open diamonds and dotted line  = A1 and; asterisks and two-dashed line  =  Clade B. For regression coefficients and associated *P-values* see Table S5.

Rapidly evolving climate niche preferences (i.e. precipitation) showed lower niche overlap (Fig 3A; Table S5) and were primarily structured by space (Fig 3A, C). The relationship between the rate of evolution and niche overlap was significant for all clades, except for a marginal effect for clade C2 (*P* = 0.06). The relationship between *Ø* and climate evolution rate was only significant for clade B (*r^2^* = 0.4, p = 0.04) and marginally significant for clades A2a and C2 (*P* = 0.07 for both clades), indicating that (except for clade C1) fast evolving climate preferences are spatially structured (Fig. 3B). In general, the relationship between niche overlap and *Ø* was negative, indicating that niche overlap is smaller for those dimensions structured in space. Note, however, that this relationship was only significant for clade A1 (*r^2^* = 0.45, p = 0.032, Fig. 3C).

### Clade diversification rate and rates of climate evolution

**Figure 4:**
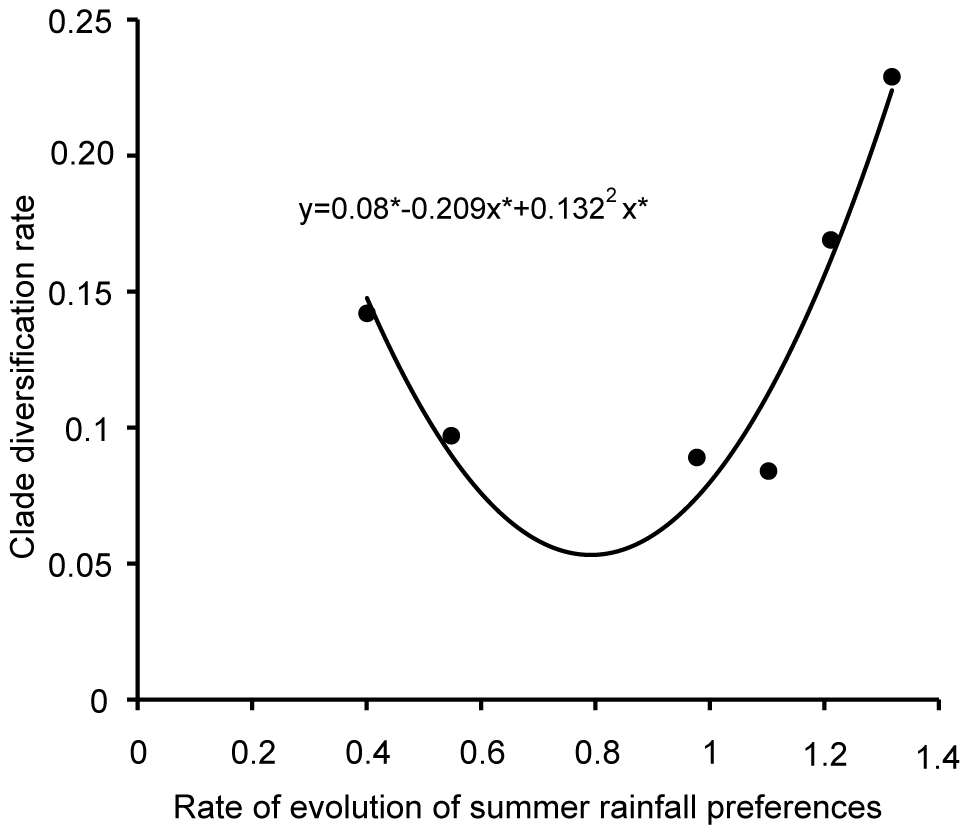
Plot showing the relationship between clade net diversification rate and rate of evolution of summer rainfall preferences (felsens). All the parameters in the polynomial regression equation are significant *P*<0.05. *r^2^* = 0.91, *P* = 0.024.

Clade diversity was positively related to clade diversification rates (*r^2^* = 0.91 p = 0.011). However these rich, rapidly diversifying clades, have experienced a tendency for decreases in diversification rates (*r^2^* = 0.77 *P* = 0.07). Clades with higher net diversification rates are younger (*r^2^* = 0.9, *P* = 0.013). Mean evolutionary rates for climate niche preferences (all climate variables) are independent of clade diversification rates (*r^2^* = 0.009, *P* = 0.85), but the latter are significantly associated with rates of summer rainfall evolution (polynomial *r^2^* = 0.915, *P* = 0.024; Fig. 4). The relatively high diversification rates of clades A2a and B are, however, not associated with high rates of summer rainfall evolution (Fig. 4).

## Discussion

We showed that rapidly evolving climate preferences were mainly driven by spatial rather than phylogenetic variation. This result indicates that the influence of recent evolutionary events rather than earlier evolutionary events were the most important in shaping species niche preferences. Our results also indicate that closely related species in the studied system have low levels of niche overlap for those climate preferences that underwent rapid evolution. In the CFR, aridification and the rapid establishment of sharp seasonal rainfall gradients were likely the events that fueled this fast evolution in climate preferences. At one end, the rapidly evolving climate preferences in *Pelargonium* is represented by summer precipitation, while, on the opposite end, preferences for energy-related variables showed greater levels of niche overlap and a greater role of phylogenetic effects, indicating evolutionary conservatism along those climate axes. This could be due to the fact that energy-related variables changed less than rainfall regimes, thus creating fewer opportunities for ecological divergence and slower evolutionary rates. High divergence in summer precipitation preferences among closely related species across *Pelargonium* clades indicates that evolvability along this niche axis is not clade dependent, although greater rates of evolution in summer precipitation were associated with larger clade sizes and diversification rates. Interestingly, some variables (e.g. winter solar radiation and summer pressure deficit) showed the reverse pattern, with greater levels of spatial structure but comparatively slow evolutionary rates. However, despite high possibilities for evolution over these climate axes, suggested by their greater spatial structuring (λ′<*Ø*), they have not resulted in significant evolutionary differentiation among closely related species. In this case, low phylogenetic signal is not correlated with higher rates of evolution, which can be expected in some evolutionary circumstances (e.g. homogeneous rate genetic drift [43]).

Higher evolutionary rates in summer precipitation preferences can be understood in light of the climatic history of the region. During the early history of *Pelargonium* (late Oligocene-Early Miocene), a summer-wet climate was present in southern Africa. The subsequent aridification, which led to the extinction of the temperate – subtropical flora that dominated the CFR landscape [44], presumably generated a new adaptive zone over which ecological specialization occurred. Indeed, most CFR (winter rainfall) *Pelargonium* lineages (A2a and A2b) experienced even greater rates of evolution in summer rainfall preferences than clades distributed in the summer rainfall region or across rainfall regimes (clades C1, C2, B). This indicates that much of the evolution of the climate niche in the genus has occurred independent of the recently created winter precipitation gradients and that pre-adaptation to summer aridity might have played an important role in diversification [14]. Diversification in *Pelargonium* has therefore mainly occurred along the increased summer aridity gradients, agreeing with extended diversification in drought adapted growth forms, leaf attributes and phenologies in the genus [22], [45]. The small chromosome clade A exhibits a much wider variation in morphological adaptations to cope with drought when compared to the large chromosome clade lineages [22]. On the other hand, the lower morphological diversity and lower diversification and climate evolutionary rates of clade C could be the result of remaining in the ancestral climate regime as evidenced by its higher diversity in the summer rainfall region. This differential capacity for diversification between major clades could also be linked to their divergent ecological strategies to cope with drought: morphologically mediated (probably throughout the development of drought avoidance, i.e. deciduousness) in clade A and through modification in their water use efficiency (development of drought resistance) in clade C [20], [46].

During the course of an adaptive radiation, a burst of diversification should be facilitated by the ecological opportunity represented by empty niches or unexploited resources, which subsequently declines as niches become filled [6], [24], [25]. Such declines in diversification rates have been observed in some groups [24], [25]. In some studies, clade age is positively related with diversity [47] but in others is instead independent (e.g. [48]). Here we found that young, highly diverse clades evolve at greater rates than old, species poor lineages. In the case of the highly diverse xerophytic clade A2b, the RC models were rejected in favor of a density dependent diversification model; this observation agrees with the significant decrease in diversification rate. Therefore for this clade, which originated about 10 MYA [13], the opening of empty niches that followed the aridification process, and its ability to evolve under increased summer drought, likely promoted an initial burst in diversification. As the radiation of this clade progressed, the niche space became saturated and the speciation rate started to decline. Niche saturation results in a decrease in opportunities for speciation [49] due to reduced chances for new populations to establish [50]. In members of clade C, we did not detect any decreases in diversification rates and thus this old radiation has diversified at constant but comparatively slower rates. In this case, the shift in climate regime did not promote an increase in diversification likely because clade C mostly diversified in the summer rainfall region, its ecological zone of origin.

As the rapid radiations of many CFR lineages have occurred *in situ* [15], the scenario of quick niche filling in the region and increased competition is highly plausible for other highly diverse, young clades (e.g. *Satyrium*, *Schoeneae*). For some of these clades increased competition, a product of niche filling, likely decreased diversification rates, yet in others might have driven diversification as suggested by phylogenetic overdispersion in some groups [51]. Phylogenetic overdispersion at the geographic scale in *Pelargonium* [20] suggests that species divergences in the genus have occurred along the new climate gradients and, as a consequence, closely related species occupy different positions along these gradients. Our findings indicate that large species and phylogenetic turnover [20] is probably the consequence of species ecological differentiation over the highly spatially structured summer precipitation gradients. It is therefore likely that the evolution of *Pelargonium* clades, especially in the CFR, was a product of the genetic isolation of ancestral populations which then diverged into new species that differentiated ecologically in response to the establishment of new summer aridity gradients. Large spatial effects in precipitation variation coupled with greater rates of evolution and low niche overlap support such a scenario. In conclusion, we suggest that *Pelargonium* diversification is the result of rapid climate change in the region associated with a fast differentiation of lineages along aridity gradients. Accelerated ecological differentiation in precipitation preferences paired with low overlap, along with diverse morphologies, suggest that the radiation in some *Pelargonium* clades (A1 and A2) was adaptive. This climate shift, along with a segmented landscape, probably contributed to the diversification of other, drought pre-adapted, hyperdiverse CFR lineages.

## Supporting Information

Figure S1 **Results of the climate niche overlap analysis for** ***Pelargonium*** **clades.**
(PDF)

Table S1 **Studied** ***Pelargonium*** **species and number of samples.**
(XLS)

Table S2 **Results of the diversification models analyses.**
(DOCX)

Table S3 **Results of climate rate of evolution analysis.**
(DOCX)

Table S4 **Results of the analysis describing the relative importance of spatial and phylogenetic effects in niche variation in** ***Pelargonium*** **clades.**
(DOCX)

Table S5 **Results of the regression analysis among evolutionary rates, niche overlap and spatial effects in** ***Pelargonium*** **clades.**
(DOCX)
