# Supplementary material for: Shifts in Climate Foster Exceptional Opportunities for Species Radiation: The Case of South African Geraniums
Source: PLoS One. 2013 Dec 17;8(12):e83087. doi: 10.1371/journal.pone.0083087 (PMC3866268; doi:10.1371/journal.pone.0083087)

Appendix S5. Results of the climate niche overlap analysis for main *Pelargonium* clades. A) Shows the mean pairwise species overlap (D) in each climate niche variable. B) Plots the proportion of rejected niche equivalency test between species pairs within clades in each climate 670 niche variable (proportion of species pairs ecologically distinct). D= Schoener niche overlap metric. MAP= mean annual precipitation; WPP= mean winter precipitation; SPP= mean summer precipitation; WVPD= mean winter pressure deficit; SVPD= mean summer pressure deficit; HU= annual heat units; WSR= mean winter solar radiation; SSR= mean summer solar radiation; PCH= accumulated positive chill units.

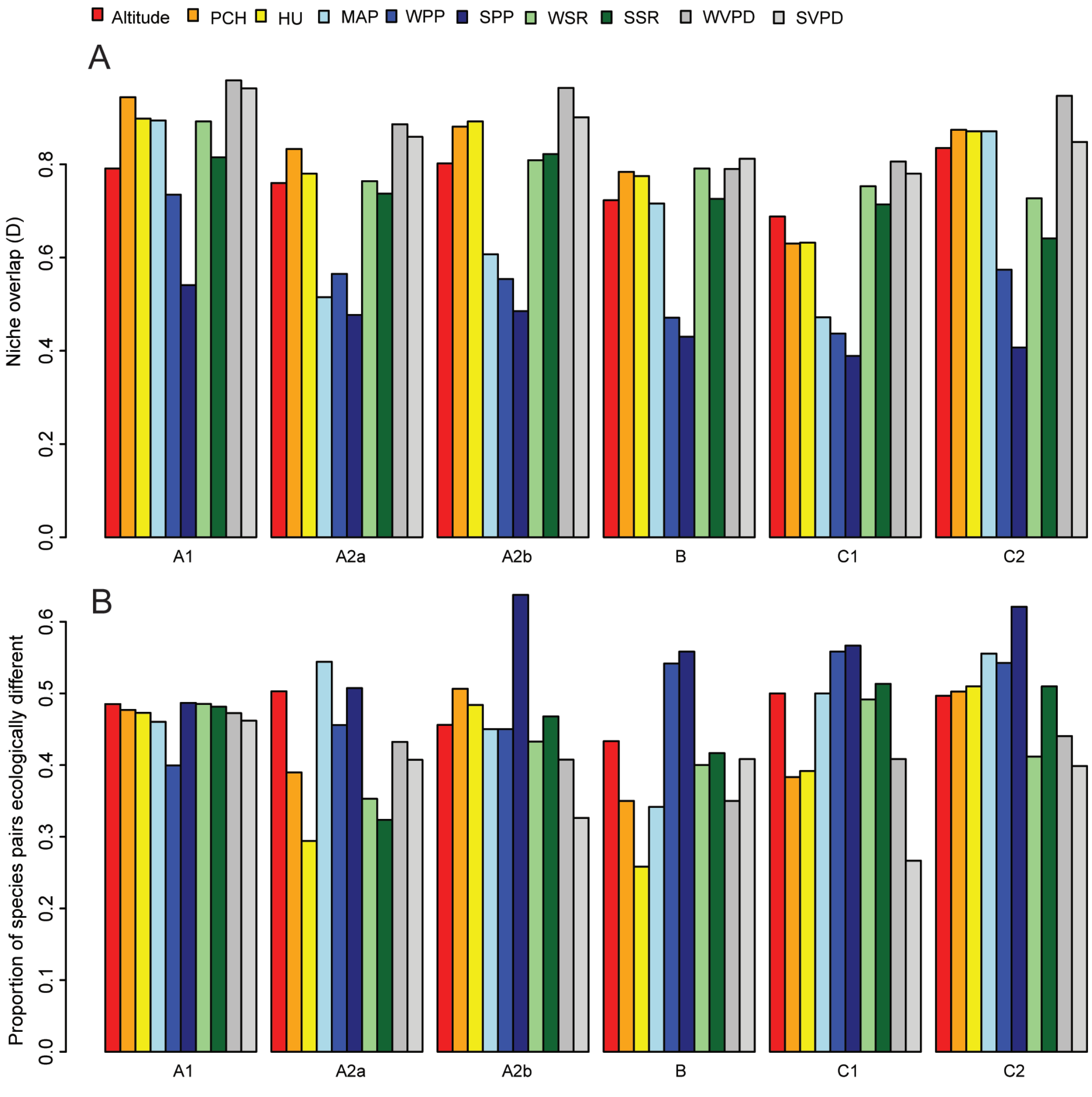

Supplement: Figure S1 — Results of the climate niche overlap analysis for Pelargonium clades. (PDF) [file pone.0083087.s001.pdf]
