# Supplementary material for: Shifts in Climate Foster Exceptional Opportunities for Species Radiation: The Case of South African Geraniums
Source: PLoS One. 2013 Dec 17;8(12):e83087. doi: 10.1371/journal.pone.0083087 (PMC3866268; doi:10.1371/journal.pone.0083087)
Supplement: Table S2 — Results of the diversification models analyses. (DOCX) [file pone.0083087.s003.docx]

**Table S2.** Results of the diversification models analyses.

| Clade A1 |  |  |  |  |
| --- | --- | --- | --- | --- |
| Model | Likelihood | AIC | ∆AIC |  |
| Pure birth | -15.5044 | 33.00881 | 0.73415 |  |
| Birth-death | -15.05518 | 34.11035 | 1.83569 | **Best RC model =Pure birth AIC=33** |
| DX | -15.40647 | 34.81294 | 2.53828 | **Best RV model = Yule-2 rate AIC=32.27** |
| DL | -15.50443 | 35.00886 | 2.7342 |  |
| Yule-2 rate | -13.13733 | 32.27466 | 0 | **∆AIC_RC_=0.73, P=0.22** |
| Yule-3 rate | -11.186 | 32.372 | 0.09734 |  |
|  |  |  |  |  |
| Clade A2a |  |  |  |  |
| Model | Likelihood | AIC | ∆AIC |  |
| Pure birth | -11.81262 | 25.62523 | 0 |  |
| Birth-death | -11.81262 | 27.62523 | 2 | **Best RC model =Pure birth AIC= 25.62** |
| DX | -11.44102 | 26.88204 | 1.25681 | **Best RV model = DDL AIC=26.14** |
| DL | -11.0728 | 26.14561 | 0.52038 |  |
| Yule-2 rate | -11.35159 | 28.70318 | 3.07795 | **∆AIC_RC_=-0.52, P=0.34** |
| Yule-3 rate | -9.168854 | 28.33771 | 2.71248 |  |
|  |  |  |  |  |
| Clade A2b |  |  |  |  |
| Model | Likelihood | AIC | ∆AIC |  |
| Pure birth | -16.29999 | 34.59998 | 7.23683 |  |
| Birth-death | -16.29999 | 36.59998 | 9.23683 | **Best RC model =Pure birth AIC= 34.59** |
| DX | -14.53196 | 33.06392 | 5.70077 | **Best RV model = DDL AIC=27.36** |
| DL | -11.68157 | 27.36315 | 0 |  |
| Yule-2 rate | -11.50155 | 29.0031 | 1.63995 | **∆AIC_RC_=7.23, P=0.007** |
| Yule-3 rate | -10.26056 | 30.52112 | 3.15797 |  |
|  |  |  |  |  |
| Clade B |  |  |  |  |
| Model | Likelihood | AIC | ∆AIC |  |
| Pure birth | -14.76413 | 31.52826 | 0.2779 |  |
| Birth-death | -14.76413 | 33.52826 | 2.2779 | **Best RC model =Pure birth AIC=31.52** |
| DX | -14.27951 | 32.55901 | 1.30865 | **Best RV model = DDL rate AIC=31.25** |
| DL | -13.62518 | 31.25036 | 0 |  |
| Yule-2 rate | -14.39464 | 34.78928 | 3.53892 | **∆AIC_RC_=0.27, P=0.22** |
| Yule-3 rate | -11.53761 | 33.07523 | 1.82487 |  |
|  |  |  |  |  |
| Clade C1 |  |  |  |  |
| Model | Likelihood | AIC | ∆AIC |  |
| Pure birth | -20.11934 | 42.23869 | 0 |  |
| Birth-death | -20.11679 | 44.23359 | 1.9949 | **Best RC model =Pure birth AIC=42.23** |
| DX | -20.08441 | 44.16882 | 1.93013 | **Best RV model =DDL AIC=43.98** |
| DL | -19.99438 | 43.98876 | 1.75007 |  |
| Yule-2 rate | -19.39099 | 44.78197 | 2.54328 | **∆AIC_RC_=-1.75, P=0.72** |
| Yule-3 rate | -17.06068 | 44.12137 | 1.88268 |  |
|  |  |  |  |  |
| Clade C2 |  |  |  |  |
| Model | Likelihood | AIC | ∆AIC |  |
| Pure birth | -15.38694 | 32.77388 | 1.5118 |  |
| Birth-death | -15.38694 | 34.77388 | 3.5118 | **Best RC model =Pure birth AIC=32.77** |
| DX | -15.21051 | 34.42102 | 3.15894 | **Best RV model =Yule-3 rate AIC=31.26** |
| DL | -14.75645 | 33.5129 | 2.25082 |  |
| Yule-2 rate | -14.26436 | 34.52871 | 3.26663 | **∆AIC_RC_=1.51, P=0.13** |
| Yule-3 rate | -10.63104 | 31.26208 | 0 |  |
