# Supplementary material for: Shifts in Climate Foster Exceptional Opportunities for Species Radiation: The Case of South African Geraniums
Source: PLoS One. 2013 Dec 17;8(12):e83087. doi: 10.1371/journal.pone.0083087 (PMC3866268; doi:10.1371/journal.pone.0083087)
Supplement: Table S3 — Results of climate rate of evolution analysis. (DOCX) [file pone.0083087.s004.docx]

**Table S3.** Results of the evolutionary rate (β) analysis of climate niche variables.

|  | Clade climate rate of evolution, β (95% CI) | | | | | |
| --- | --- | --- | --- | --- | --- | --- |
| Climate niche variable | A1 | A2a | A2b | B | C1 | C2 |
| Accumulated positive chill units | 0.131 (0.05-0.26) | 0.297 (0.11-0.58) | 0.24 (0.094-0.47) | 0.100 (0.039-0.19) | 0.214 (0.084-0.42) | 0.210 (0.08-0.04) |
| Altitude (m) | 0.311 (0.12-0.61) | 0.245 (0.09-0.48) | 0.195 (0.07-0.38) | 0.215 (0.084-0.42) | 0.177 (0.07-0.35) | 0.339 (0.13-0.67) |
| Winter solar radiation (MJ · m− 2 · d− 1) | 0.010 (0.004-0.02) | 0.007 (0.002-0.013) | 0.007 (0.002-0.013) | 0.012 (0.004-0.023) | 0.013 (0.005-0.026) | 0.006 (0.002-0.012) |
| Summer solar radiation (MJ · m− 2 · d− 1) | 0.003 (0.001-0.005) | 0.005 (0.002-0.009) | 0.004 (0.001-0.008) | 0.004 (0.001-0.008) | 0.002 (0.0009-0.004) | 0.020 (0.007-0.039) |
| Winter vapor pressure deficit (mm) | 0.208 (0.08-0.41) | 0.060 (0.02-0.12) | 0.059 (0.023-0.11) | 0.048 (0.019-0.095) | 0.053 (0.021-0.0106) | 0.130 (0.051-0.25) |
| Summer vapor pressure deficit (mm) | 0.066 (0.026-0.13) | 0.069 (0.027-0.137) | 0.059 (0.023-0.11) | 0.218 (0.086-0.43) | 0.054 (0.021-0.0107 | 0.195 (0.07-0.38) |
| Winter precipitation (mm) | 0.553 (0.21-1.09) | 0.407 (0.16- 0.8) | 0.396 (0.15-0.78) | 0.267 (0.1-0.53) | 0.506 (0.2-1) | 0.427 (0.17-0.84) |
| Summer precipitation (mm) | 0.401 (0.15-0.79) | 1.211 (0.48-2.4) | 1.318 (0.52-2.61) | 0.548 (0.21-1.089) | 1.102 (0.43-2.19) | 0.977 (0.38-1.94) |
| Mean annual precipitation (mm) | 0.129 (0.05-0.25) | 0.202 (0.08-0.4) | 0.210 (0.083-0.42) | 0.154 (0.06-0.3) | 0.189 (0.075-0.37) | 0.131 (0.05-0.26) |
| Heat units (˚d) | 0.029 (0.01-0.6) | 0.018 (0.007-0.35) | 0.023 (0.009-0.045) | 0.009 (0.003-0.018) | 0.024 (0.009-0.047) | 0.036 (0.014-0.07) |
| Clade mean β | 0.184 | 0.252 | 0.251 | 0.157 | 0.233 | 0.247 |
