# Supplementary material for: Shifts in Climate Foster Exceptional Opportunities for Species Radiation: The Case of South African Geraniums
Source: PLoS One. 2013 Dec 17;8(12):e83087. doi: 10.1371/journal.pone.0083087 (PMC3866268; doi:10.1371/journal.pone.0083087)
Supplement: Table S4 — Results of the analysis describing the relative importance of spatial and phylogenetic effects in niche variation in Pelargonium clades. (DOCX) [file pone.0083087.s005.docx]

**Table S4.** Parameters describing spatial and phylogenetic effects in niche variation in main clades. Maximum likelihood values of the parameters Ø and λ , composite parameters λ′ (spatially corrected phylogenetic signal) Γ (climate variation independent from spatial and phylogenetic effects). L= maximum likelihood.

| **A1** |  |  |  | Composite parameters | |  |
| --- | --- | --- | --- | --- | --- | --- |
|  | *Ø* | λ | *L* | Γ | λ′ | *Ø* |
| Mean annual precipitation (mm) | 0.474 | 0.010 | -184.631 | 0.521 | 0.005 | 0.474 |
| Heat units (˚d) | 0.457 | 0.010 | -205.285 | 0.538 | 0.005 | 0.457 |
| Summer precipitation (mm) | 1.000 | n.a. | -97.549 | 0 | 0 | 1.000 |
| Winter precipitation (mm) | 0.739 | 0.010 | -136.610 | 0.259 | 0.003 | 0.739 |
| Summer vapor pressure deficit (mm) | 0.022 | 0.010 | -130.704 | 0.968 | 0.010 | 0.022 |
| Winter vapor pressure deficit (mm) | 0.147 | 0.010 | -134.517 | 0.844 | 0.009 | 0.147 |
| Summer solar radiation ((MJ · m− 2 · d− 1) | 0.371 | 0.010 | -41.196 | 0.623 | 0.006 | 0.371 |
| Winter vapor pressure deficit (mm) | 0.952 | 0.010 | -27.620 | 0.048 | 0.000 | 0.952 |
| Accumulated positive chill units | 0.650 | 0.010 | -201.921 | 0.346 | 0.003 | 0.650 |
| Altitude (m) | 0.841 | 0.010 | -197.232 | 0.157 | 0.002 | 0.841 |
| Mean |  |  |  | 0.478 | 0.005 | 0.565 |
|  |  |  |  |  | |  |
| **A2A** |  |  |  | Composite parameters | |  |
|  | *Ø* | λ | *L* | Γ | λ′ | *Ø* |
| Mean annual precipitation (mm) | 0.660 | 0.010 | -93.191 | 0.337 | 0.003 | 0.660 |
| Heat units (˚d) | 0.010 | 0.287 | -108.776 | 0.706 | 0.284 | 0.010 |
| Summer precipitation (mm) | 0.947 | 0.010 | -59.187 | 0.053 | 0.001 | 0.947 |
| Winter precipitation (mm) | 0.508 | 0.010 | -60.611 | 0.487 | 0.005 | 0.508 |
| Summer vapor pressure deficit (mm) | 0.718 | 0.010 | -70.071 | 0.279 | 0.003 | 0.718 |
| Winter vapor pressure deficit (mm) | 0.446 | 0.010 | -67.869 | 0.548 | 0.006 | 0.446 |
| Summer solar radiation ((MJ · m− 2 · d− 1) | 0.010 | 0.990 | -27.917 | 0.010 | 0.980 | 0.010 |
| Winter vapor pressure deficit (mm) | 0.842 | 0.010 | -18.868 | 0.156 | 0.002 | 0.842 |
| Accumulated positive chill units | 0.010 | 0.010 | -106.936 | 0.980 | 0.010 | 0.010 |
| Altitude (m) | 0.010 | 0.010 | -103.015 | 0.980 | 0.010 | 0.010 |
| Mean |  |  |  | 0.454 | 0.130 | 0.416 |
|  |  |  |  |  | |  |
| **A2B** |  |  |  | Composite parameters | |  |
|  | *Ø* | λ | *L* | Γ | λ′ | *Ø* |
| Mean annual precipitation (mm) | 0.708 | 0.010 | -110.944 | 0.289 | 0.003 | 0.708 |
| Heat units (˚d) | 0.423 | 0.990 | -126.003 | 0.006 | 0.571 | 0.423 |
| Summer precipitation (mm) | 0.990 | 0.010 | -72.902 | 0.010 | 0.000 | 0.990 |
| Winter precipitation (mm) | 0.282 | 0.010 | -75.722 | 0.710 | 0.007 | 0.282 |
| Summer vapor pressure deficit (mm) | 0.772 | 0.010 | -82.042 | 0.225 | 0.002 | 0.772 |
| Winter vapor pressure deficit (mm) | 0.010 | 0.781 | -76.911 | 0.217 | 0.773 | 0.010 |
| Summer solar radiation ((MJ · m− 2 · d− 1) | 0.542 | 0.010 | -29.457 | 0.453 | 0.005 | 0.542 |
| Winter vapor pressure deficit (mm) | 0.968 | 0.010 | -21.897 | 0.031 | 0.000 | 0.968 |
| Accumulated positive chill units | 0.010 | 0.700 | -121.358 | 0.297 | 0.693 | 0.010 |
| Altitude (m) | 0.584 | 0.010 | -118.918 | 0.412 | 0.004 | 0.584 |
| Mean |  |  |  | 0.265 | 0.206 | 0.529 |
|  |  |  |  |  | |  |
| **B** |  |  |  | Composite parameters | |  |
|  | *Ø* | λ | *L* | Γ | λ′ | *Ø* |
| Mean annual precipitation (mm) | 0.990 | 0.010 | -87.427 | 0.010 | 0.000 | 0.990 |
| Heat units (˚d) | 0.010 | 0.010 | -92.318 | 0.980 | 0.010 | 0.010 |
| Summer precipitation (mm) | 0.990 | 0.010 | -65.270 | 0.010 | 0.000 | 0.990 |
| Winter precipitation (mm) | 0.990 | 0.010 | -53.555 | 0.010 | 0.000 | 0.990 |
| Summer vapor pressure deficit (mm) | 0.990 | 0.010 | -60.860 | 0.010 | 0.000 | 0.990 |
| Winter vapor pressure deficit (mm) | 0.470 | 0.010 | -60.560 | 0.525 | 0.005 | 0.470 |
| Summer solar radiation ((MJ · m− 2 · d− 1) | 0.031 | 0.010 | -26.252 | 0.960 | 0.010 | 0.031 |
| Winter vapor pressure deficit (mm) | 0.871 | 0.010 | -20.905 | 0.128 | 0.001 | 0.871 |
| Accumulated positive chill units | 0.208 | 0.010 | -91.126 | 0.784 | 0.008 | 0.208 |
| Altitude (m) | 0.737 | 0.010 | -97.607 | 0.260 | 0.003 | 0.737 |
| Mean |  |  |  | 0.368 | 0.004 | 0.629 |
|  |  |  |  |  | |  |
| **C1** |  |  |  | Composite parameters | |  |
|  | *Ø* | λ | *L* | Γ | λ′ | *Ø* |
| Mean annual precipitation (mm) | 0.990 | 0.010 | -89.299 | 0.010 | 0.000 | 0.990 |
| Heat units (˚d) | 0.990 | 0.010 | -105.813 | 0.010 | 0.000 | 0.990 |
| Summer precipitation (mm) | 0.990 | 0.010 | -63.216 | 0.010 | 0.000 | 0.990 |
| Winter precipitation (mm) | 0.325 | 0.010 | -60.926 | 0.668 | 0.007 | 0.325 |
| Summer vapor pressure deficit (mm) | 0.990 | 0.010 | -69.959 | 0.010 | 0.000 | 0.990 |
| Winter vapor pressure deficit (mm) | 0.990 | 0.010 | -68.224 | 0.010 | 0.000 | 0.990 |
| Summer solar radiation ((MJ · m− 2 · d− 1) | 0.990 | 0.010 | -22.852 | 0.010 | 0.000 | 0.990 |
| Winter vapor pressure deficit (mm) | 0.990 | 0.010 | -24.592 | 0.010 | 0.000 | 0.990 |
| Accumulated positive chill units | 0.971 | 0.010 | -98.330 | 0.029 | 0.000 | 0.971 |
| Altitude (m) | 0.971 | 0.010 | -97.595 | 0.029 | 0.000 | 0.971 |
| Mean |  |  |  | 0.079 | 0.001 | 0.920 |
|  |  |  |  |  |  |  |
| **C2** |  |  |  | Composite parameters | |  |
|  | *Ø* | λ | *L* | Γ | λ′ | *Ø* |
| Mean annual precipitation (mm) | 0.944 | 0.010 | -104.300 | 0.056 | 0.001 | 0.944 |
| Heat units (˚d) | 0.709 | 0.010 | -126.005 | 0.288 | 0.003 | 0.709 |
| Summer precipitation (mm) | 0.990 | 0.010 | -77.668 | 0.010 | 0.000 | 0.990 |
| Winter precipitation (mm) | 0.880 | 0.010 | -68.851 | 0.118 | 0.001 | 0.880 |
| Summer vapor pressure deficit (mm) | 0.825 | 0.010 | -81.619 | 0.173 | 0.002 | 0.825 |
| Winter vapor pressure deficit (mm) | 0.370 | 0.010 | -84.352 | 0.624 | 0.006 | 0.370 |
| Summer solar radiation ((MJ · m− 2 · d− 1) | 0.559 | 0.010 | -33.991 | 0.437 | 0.004 | 0.559 |
| Winter vapor pressure deficit (mm) | 0.358 | 0.010 | -31.120 | 0.636 | 0.006 | 0.358 |
| Accumulated positive chill units | 0.268 | 0.010 | -114.561 | 0.725 | 0.007 | 0.268 |
| Altitude (m) | 0.954 | 0.010 | -115.934 | 0.046 | 0.000 | 0.954 |
| Mean |  |  |  | 0.311 | 0.003 | 0.686 |
