# Supplementary material for: Shifts in Climate Foster Exceptional Opportunities for Species Radiation: The Case of South African Geraniums
Source: PLoS One. 2013 Dec 17;8(12):e83087. doi: 10.1371/journal.pone.0083087 (PMC3866268; doi:10.1371/journal.pone.0083087)
Supplement: Table S5 — Results of the regression analysis among evolutionary rates, niche overlap and spatial effects in Pelargonium clades. (DOCX) [file pone.0083087.s006.docx]

**Table S5.** Results of the regression analysis (R^2^ and P-value) among evolutionary rates (felsens), niche overlap (D) and spatial effects (*Ø*) in main *Pelargonium* clades.

| Clade A1 |  |  |
| --- | --- | --- |
|  | felsens | D |
| D | -0.43(0.03) |  |
| *Ø* | 0.17 (0.22) | 0.45 (0.032) |
|  |  |  |
| Clade A2a |  |  |
|  | felsens | D |
| D | -0.47(0.02) |  |
| *Ø* | 0.18 (0.07) | 0.18 (0.21) |
|  |  |  |
| Clade A2b |  |  |
|  | felsens | D |
| D | -0.43(0.03) |  |
| *Ø* | 0.11(0.34) | 0.11 (0.32) |
|  |  |  |
| Clade B |  |  |
|  | felsens | D |
| D | -0.67(0.003) |  |
| *Ø* | 0.4 (0.04) | 0.2 (0.18) |
|  |  |  |
| Clade C1 |  |  |
|  | felsens | D |
| D | -0.63 (0.005) | |
| *Ø* | -0.07 (0.43) | 0.207 (0.18) |
|  |  |  |
| Clade C2 |  |  |
|  | felsens | D |
| D | -0.4(0.06) |  |
| *Ø* | 0.38(0.07) | 0.11 (0.36) |
